# Assessment of Risk Factors for Conversion in Laparoscopic Cholecystectomy Performed Due to Symptomatic Cholecystolithiasis

**DOI:** 10.7759/cureus.84326

**Published:** 2025-05-18

**Authors:** Bartosz Molasy, Mateusz Frydrych, Aldona Kubala-Kukus, Kamil Nieroda, Stanislaw Gluszek

**Affiliations:** 1 Department of General Surgery, Jan Kochanowski University, Kielce, POL; 2 Department of General Surgery, St. Alexander Hospital, Kielce, POL; 3 Institute of Physics, Jan Kochanowski University, Kielce, POL; 4 School of Medicine, Jan Kochanowski University, Kielce, POL; 5 Department of Surgical Oncology, Holy Cross Cancer Center, Kielce, POL

**Keywords:** cholelithiasis, conversion, enhanced recovery after surgery, eras, laparoscopic cholecystectomy, risk factor

## Abstract

Introduction: Laparoscopic cholecystectomy is the current gold standard in the treatment of symptomatic cholecystolithiasis. The occurrence of the conversion is related to higher perioperative mortality or prolonged hospitalization. The aim of this study was to evaluate selected risk factors for conversion of laparoscopic cholecystectomy among patients undergoing surgery due to symptomatic cholelithiasis.

Patients and methods: A retrospective analysis of patients operated on for symptomatic cholelithiasis from November 2021 to June 2023 was performed. Correlations between selected factors and the occurrence of conversion were analyzed. Early outcomes of surgical treatment and the impact of using the Enhanced Recovery After Surgery (ERAS) protocol were analyzed.

Results: The analysis concerned 227 patients who were operated on due to symptomatic cholelithiasis. In 28 (12.3%) cases, the procedure was converted to an open method. A multivariate analysis showed that male gender (p=0.003, OR=0.196), type 2 diabetes (p=0.018, OR=4.045), older age (p=0.008, OR=1.063), and a history of acute cholecystitis (p<0.0001, OR=1.981) predispose to conversion. The occurrence of conversion is associated with the increased duration of surgery and hospitalization, a higher rate of surgical site infections, and clinically significant complications (p<0.0001). Statistically significant differences were found in the length of stay depending on the use of the ERAS protocol (p<0.0001).

Conclusions: The most important preoperative risk factors for conversion in the case of cholecystectomy performed due to symptomatic cholelithiasis include male gender, older age, type 2 diabetes, and a history of an episode of acute cholecystitis. The use of the ERAS protocol is safe and does not increase the conversion rate or clinically significant postoperative complications.

## Introduction

Cholelithiasis is one of the most common diseases requiring surgical treatment. Laparoscopic cholecystectomy (LC) is the current gold standard in the treatment of this disease [1]. According to available literature, the cholecystectomy is converted to an open method in 5-15% of cases [1,2]. The main reasons for conversion include the inability to obtain a critical view of safety or intraoperative complications such as hemorrhage or iatrogenic injury of the biliary tree [3]. Risk factors for conversion include age, gender, type of admission, or comorbidities [1,2,4]. The use of the Enhanced Recovery After Surgery (ERAS) protocol can reduce the rate of postoperative complications in many surgical specialties [5]. There have been many reports on the use of ERAS in surgery, but only a few studies have analyzed the usefulness of ERAS in LC; therefore, this clinical aspect requires analysis and further investigation.

Determining preoperative factors affecting the conversion rate in the case of cholecystectomy is crucial. It allows for the identification of a group with a high risk of conversion, which thus allows for better preparation for the surgery and safer performance [3]. The occurrence of conversion is related to higher perioperative mortality or prolonged hospitalization [1,4]. Therefore, determining the group at high risk of conversion is important and should be as simple as possible so that it can be used widely in everyday surgical practice.

This study aimed to evaluate selected risk factors for converting cholecystectomy from a laparoscopic to an open method among patients undergoing surgery due to symptomatic cholelithiasis.

## Materials and methods

A retrospective analysis of 245 patients operated on for symptomatic cholelithiasis from November 2021 to June 2023 was performed. After meeting the inclusion and exclusion criteria, 227 were included in the study.

The following inclusion criteria were defined as age ≥ 18 years, presence of symptomatic cholelithiasis confirmed by imaging tests, and availability of documentation containing complete analyzed data. The exclusion criteria were qualification for cholecystectomy surgery for an indication other than symptomatic cholelithiasis and performing surgery primarily using the open method.

The analyzed data included age, gender, type of admission, comorbidities, American Society of Anesthesiologists (ASA) grade, history of episodes of acute cholecystitis (AC), regardless of the time interval prior to surgery and acute pancreatitis (AP), history of the endoscopic retrograde cholangiopancreatography (ERCP) or surgical procedures, and the operator's experience. Early outcomes of surgical treatment were also analyzed in the study population, including the duration of the surgical procedure, length of hospital stay, occurrence of surgical site infection, and postoperative complications determined in the Clavien-Dindo scale. Operative time was defined as the duration from initial skin incision to final skin closure, regardless of the surgical approach. In patients who underwent conversion, the time includes both the laparoscopic and open phases of the procedure.

In addition, the impact of the ERAS protocol on early outcomes was also analyzed in the study population. Our institution now follows a standardized ERAS protocol, including preoperative carbohydrate loading, no routine use of drains, multimodal analgesia, and early oral intake. All clinical and procedural data were independently reviewed by two surgical residents and verified by a senior attending surgeon.

Due to significant deviations from the normal distribution of quantitative variables, assessed with the Shapiro-Wilk test and graphically with a Q-Q chart, non-parametric methods were used. Collected data were presented as medians with interquartile ranges for quantitative variables and number of cases and percentage for qualitative variables. The Mann-Whitney U test was used to compare quantitative variables. The chi-square test was used to compare relationships in qualitative variables. Correlations were assessed using Spearman's rank correlation coefficient. A logistic regression model was made to assess the risk of conversion, where p<0.05 was considered statistically significant.

All procedures performed were in accordance with the 1964 Helsinki Declaration and its later amendments. Our study received positive approval from the Bioethics Committee of the Jan Kochanowski University Medical College (approval no. 1/2024).

## Results

The analysis concerned 227 patients operated on due to symptomatic cholelithiasis. In 28 (12.3%) cases, the procedure was converted to an open method. The study group included 61 (26.9%) men and 166 (73.1%) women. Conversion occurred more often among men than women (24.6% vs. 7.8%, p = 0.0007). In 206 (90.75%) cases, procedures were performed electively, whereas 21 (9.25%) cases were performed urgently.

In the studied population, the most common comorbidities were hypertension, obesity, and type 2 diabetes. Other diseases occurred less frequently. In 19 (11.5%) cases, patients were diagnosed with multimorbidity (at least three chronic diseases). Based on the analysis, it was found that conversion occurred statistically significantly more often among patients diagnosed with obesity, hypertension, heart failure, ischemic heart disease, type 2 diabetes, and multimorbidity (p<0.05). It was found that the percentage of conversions for ASA Grades I and II is equal in the compared groups, but it is statistically significantly higher in the case of ASA Grade III (p<0.0001). In the analyzed group, there were no ASA grades IV or V.

A significant increase in the conversion rate was observed in patients who had AC <6 weeks before surgery and those operated on in the acute phase of inflammation (44.4% and 55.0%, respectively). These were statistically significant differences (p<0.01). There were no statistically significant differences in the conversion frequency between the group of patients with AP and those without a history of AP (p>0.05).

Fifteen (6.6%) patients underwent ERCP due to choledocholithiasis. Furthermore, 74 patients (32.6%) had a history of previous abdominal surgery, with only one (0.1%) case having a history of surgery in the upper abdominal cavity. In the remaining cases, these were surgical procedures affecting the lower abdominal cavity. There were no statistically significant differences in the conversion rate if the patient had previously undergone ERCP or previous abdominal surgery (p>0.05). In the analyzed group, conversions occurred more often in cases where the operator was a resident compared to the group of patients operated on by a specialist (18.6% and 10.9%, respectively). However, this difference was not statistically significant (p>0.05). The relationships described above are presented in Table 1.

**Table 1:** Clinical characteristics of the analyzed population and univariate analyses of conversion risk factors P, statistical significance level; COPD, chronic obstructive pulmonary disease; ASA, American Society of Anesthesiologists; ERCP, endoscopic retrograde cholangiopancreatography

Variable		N (total)	Group without conversion	Group with conversion	P-value
		227 (100%)	199 (87.7%)	28 (12.3%)	
Gender	Men	61 (26.9%)	46 (75.4%)	15 (24.6%)	0.0007
	Women	166 (73.1%)	153 (92.2%)	13 (7.8%)	
Age	Mean	54.9	53.3	67	<0.0001
	Median	58	54	68.5	
Type of admission	Elective	206 (90.8%)	188 (91.3%)	18 (8.7%)	<0.0001
	Emergency	21 (9.2%)	11 (52.4%)	10 (47.6%)	
Obesity	No	163 (71.8%)	151 (92.4%)	12 (7.4%)	0.0003
	Yes	64 (28.2%)	48 (75.0%)	16 (25.0%)	
Hypertension	No	121 (53.3%)	115 (95.0%)	6 (5.0%)	0.0002
	Yes	106 (46.7%)	84 (79.2%)	22 (20.8%)	
Heart failure	No	221 (97.3%)	196 (88.7%)	25 (11.3%)	0.0045
	Yes	6 (2.7%)	3 (50.0%)	3 (50.0%)	
Ischemic heart disease	No	217 (95.6%)	193 (88.9%)	24 (11.1%)	0.0065
	Yes	10 (4.4%)	6 (60.0%)	4 (40.0%)	
Type 2 diabetes	No	193 (85.0%)	177 (91.7%)	16 (8.3%)	<0.0001
	Yes	34 (15.0%)	22 (64.7%)	12 (35.3%)	
Asthma/COPD	No	219 (96.5%)	192 (87.7%)	27 (12.3%)	>0.05
	Yes	8 (3.5%)	7 (87.5%)	1 (12.5%)	
Chronic kidney disease	No	223 (98.2%)	196 (87.9%)	27 (12.1%)	>0.05
	Yes	4 (1.8%)	2 (50.0%)	2 (50.0%)	
Multimorbidity	No	208 (91.6%)	189 (90.9%)	19 (9.1%)	<0.0001
	Yes	19 (8.4%)	10 (52.6%)	9 (47.4%)	
ASA grade	I	34 (15.0%)	34 (100.0%)	0	<0.0001
	II	171 (75.3%)	157 (91.8%)	14 (8.2%)	
	III	22 (9.7%)	8 (36.4%)	14 (63.6%)	
An episode of acute cholecystitis	No	179 (78.9%)	169 (94.4%)	10 (5.6%)	<0.01
	Yes. surgery in the acute phase	20 (8.8%)	9 (45.0%)	11 (55.0%)	
	Yes. <6 weeks	9 (4.0%)	5 (55.6%)	4 (44.4%)	
	Yes. >6 weeks	19 (8.4%)	16 (84.2%)	3 (15.8%)	
Acute pancreatitis caused by gallstones in the past	No	216 (95.1%)	189 (87.5%)	27 (12.5%)	>0.05
	Yes	11 (4.9%)	10 (91.0%)	1 (9.0%)	
Status after ERCP	No	212 (93.4%)	187 (88.2%)	25 (11.8%)	>0.05
	Yes	15 (6.6%)	12 (80.0%)	3 (20.0%)	
Previous abdominal surgery	No	153 (67.4%)	132 (86.3%)	21 (13.7%)	>0.05
	Yes	74 (32.6%)	67 (90.5%)	7 (9.5%)	
Surgeon experience	Resident	43 (18.9%)	35 (81.4%)	8 (18.6%)	>0.05
	Specialist	184 (81.1%)	164 (89.1%)	20 (10.9%)	

**Figure 1:**
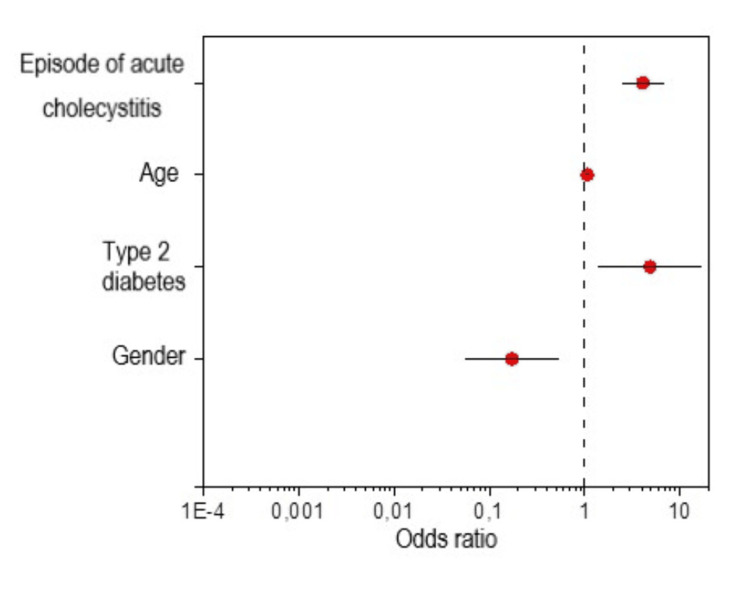
Forest plot for a multivariate conversion risk model

A multivariate analysis of conversion risk factors was also performed in the study population. On its basis, a statistical model was created according to which male gender, type 2 diabetes, older age, and an episode of AC in the past predispose to conversion of cholecystectomy from the minimally invasive to the open method. Male gender is a risk factor for conversion (p=0.003, OR=0.196). The occurrence of type 2 diabetes increases this risk four times (p=0.018, OR=4.045), and a unit increase in age increases the risk of conversion approximately 1.06 times (p=0.008, OR=1.063). In turn, a unit increase in the value of the variable defined as the occurrence of AC in the obtained statistical model increases the risk of converting a laparoscopic procedure to an open procedure almost twice (p<0.0001, OR=1.981). The statistical model is presented in Table 2 and Figure 1.

**Table 2:** Multivariate model for conversion risk P, statistical significance level; OR, odds ratio; CI, confidence interval

Variable	P-value	OR	95% CI for OR (lower/upper bound)
Gender (1 - men, 2 - women)	0.003	0.173	0.055/0.544
Type 2 diabetes	0.013	4,893	1,402/17,071
Age	0.005	1,071	1,021/1,124
Episode of acute cholecystitis (0 - absence, 1 - >6 weeks, 2 - <6 weeks, 3 - surgery in the acute phase)	<0.0001	4,092	2,448/6,841

In the group of patients operated on without conversion, the duration of the procedure was longer (median 70 minutes) compared to the group with conversion (median 105 minutes), and the difference was statistically significant (p<0.0001). In the analyzed groups, statistically significant differences were also found in the length of hospital stay (p<0.0001). All six cases (2.6%) of surgical site infections occurred in the conversion group (p < 0.0001). Based on the analysis, it was also found that the severity of complications according to the Clavien-Dindo scale depends on the occurrence of conversion (p<0.0001). Clinically significant complications (Grade III and higher) occurred four times more often in the group of patients with conversion. The relationships described above are presented in Table 3.

**Table 3:** Early outcomes in the analyzed population P, statistical significance level.

Variable		N (total)	Group without conversion	Group with conversion	P-value
		227 (100%)	199 (87.7%)	28 (12.3%)	
Duration of the procedure (minutes)	Mean	76.3	71.1	113.9	<0.0001
	Median	70	70	105	
Length of hospitalization (days)	Median	2	2	4	<0.0001
Surgical site infection	No	221 (97.4%)	199 (90.0%)	22 (10.0%)	<0.0001
	Yes	6 (2.6%)	0 (0.0%)	6 (100.0%)	
The grade of postoperative complications according to the Clavien-Dindo scale	0	166 (73.1%)	162 (97.6%)	4 (2.4%)	<0.0001
	I	27 (11.8%)	21 (77.8%)	6 (22.2%)	
	II	26 (11.5%)	14 (53.8%)	12 (46.2%)	
	III	8 (3.5%)	2 (25.0%)	6 (75.0%)	
	IV	0	0	0	
	V	0	0	0	

The ERAS protocol was used in 121 (53.3%) cases. No statistically significant differences were found in the duration of the surgical procedure between the group of patients in whom the ERAS protocol was not used and those in whom it was used (p=0.44). The relationship between the length of stay and the use of the ERAS protocol was also analyzed. Statistically significant differences were found in the length of stay depending on the use of the protocol (p<0.0001). The relationships described above are presented in Table 4.

**Table 4:** Duration of surgery and length of stay depending on the use of the ERAS protocol ERAS, Enhanced Recovery After Surgery

Variable	Without ERAS (N=106)			With ERAS (N=121)			P-value
	Mean	Median	Min/max	Mean	Median	Min/max	
Duration of the procedure (minutes)	76.6	70.0	35.0/190.0	76.0	70.0	40.0/175.0	0.44
Length of stay (days)	2.9	2.0	1.0/29.0	2.2	2.0	1.0/24.0	<0.0001

The incidence of surgical site infection was analyzed in relation to the use of the ERAS protocol. It was found that the incidence of surgical site infection decreased when the protocol was used (rS=-0.121). However, this was not a statistically significant relationship (p>0.05). The relationship between the grade of postoperative complications according to the Clavien-Dindo scale and the use of the ERAS protocol was also analyzed. It was found that the severity of postoperative complications assessed in the Clavien-Dindo scale decreased when the ERAS protocol was used (rS=-0.165). However, this relationship was not statistically significant in this case either (p>0.05).

## Discussion

Cholelithiasis is a common disease that requires surgical treatment. The gold standard surgical treatment of symptomatic cholelithiasis is LC [1]. According to available literature, conversion of the cholecystectomy from laparoscopic to open method occurs in 5-15% of cases [1,2,6]. In the analyzed population, the procedure was converted to an open method in 28 (12.3%) cases.

Cholelithiasis is four times more common in women than in men [7], which is also reflected in the analyzed population, where women prevailed (73.1% of cases). However, conversion occurred three times more often in men than in women (p = 0.0007). Many reports suggest that male gender is a risk factor for conversion [4,8,9]. This is due to the fact that in men, compared to women, there are more frequent episodes of AC, which is connected with a greater number of inflammatory adhesions that complicate the surgical procedure [10]. Another reason for the higher percentage of conversions among men is the fact that they consult a doctor later after the onset of symptoms of cholelithiasis, at a more advanced stage of the disease than women [9]. Compared to previously published cohorts, our population had a slightly higher male proportion and a higher frequency of prior AC. These factors may have influenced the conversion rate and highlight the importance of demographic context in interpreting surgical risk. The generalizability of our findings remains limited to similar settings and warrants multicenter validation.

With increasing age, the frequency of conversion from laparoscopic to open surgery increases [1,2,4,6,11]. A similar relationship was confirmed in the study population (OR 1.071, p=0.005). The analysis found that conversion occurs statistically significantly more often in older patients (p<0.0001). The rate of conversion is also influenced by the mode in which the patient is operated on. Emergency procedures are associated with a higher conversion rate compared to elective procedures [1,6,10,12]. Also in the study population, a higher conversion rate was found among patients operated on for urgent indications (p<0.0001).

The analyzed literature reports that some chronic diseases are a risk factor for conversion in the case of cholecystectomy. These mainly include obesity [3,13], type 2 diabetes [3,6,14,15], or cardiac diseases [10,13]. According to Al Masri et al., pulmonary diseases are also a risk factor for conversion [10]. Some analyses state that obesity is not a risk factor for conversion in LC [16,17]. In the analyzed population, it was found that obesity, hypertension, heart failure, ischemic heart disease, and type 2 diabetes are risk factors for conversion (p<0.05). Comorbidities and the state of their compensation affect the ASA score determined before surgery. The analyzed literature revealed reports according to which the ASA grade, defined as Grade III and higher, increases the frequency of LC conversion [3,10]. A similar relationship was confirmed during this analysis.

Cholecystectomy performed due to AC increases the risk of conversion [6,18,19]. Also in the analyzed population, it was found that the highest conversion rate (55.0%) occurred in patients operated on for AC (p<0.01). According to the guidelines of the World Society of Emergency Surgery, if early cholecystectomy cannot be performed (up to seven days from the onset of symptoms), delayed cholecystectomy should be performed after a minimum of six weeks due to the increased risk of postoperative complications and higher perioperative mortality if the procedure is performed during the first six weeks after the onset of an episode of AC [20]. The occurrence of an episode of AC below three weeks before surgery significantly increases the conversion rate [3]. In this analysis, statistically significant differences were found in the percentage of conversion between the group of patients in whom cholecystitis occurred less than six weeks before surgery (44.4%) and the group of patients with an episode of AC more than six weeks before surgery (15.8%) (p<0.01). In turn, between the group of patients with an episode of AC lasting longer than six weeks and the group of patients without an episode of cholecystitis in their history, no statistically significant differences were found in the frequency of conversion.

Based on the analyzed literature, a past episode of AP caused by gallstones is not a risk factor for conversion [1,3,6]. Also in this analysis, the influence of AP on the incidence of conversion was not confirmed (p>0.05). In turn, the impact of ERCP in the past on the risk of conversion remains a matter of debate. According to some authors, a history of ERCP increases the risk of conversion in the case of LC [1,13,18,21]. However, there are reports that post-ERCP status is not a risk factor for conversion [6,10,14]. This analysis did not confirm the relationship between ERCP and an increased conversion rate (p>0.05).

In the case of a history of abdominal surgery, most authors believe that this is a risk factor for conversion [3,10,13,21]. However, in most analyses, only a history of upper abdominal surgery increases the risk of conversion [3,10,21], even though lower abdominal surgery in the past does not constitute a risk factor [3,10,21]. In the analyzed population, there was no relationship between a history of surgery and an increased conversion rate (p>0.05). This is due to the fact that out of 74 patients (32.6%), only one (0.1%) had previous surgical procedures in the upper abdominal cavity.

In the reviewed literature, some analyses concerned the influence of operator experience on the conversion rate in LC [17,21]. According to Ochoa-Ortiz et al., experienced laparoscopic surgeons are more likely to complete the procedure with a minimally invasive method in cases of difficult cholecystectomy [17]. In turn, the analysis by Abraham et al. showed that as the operator's experience increases, the percentage of patients who undergo conversion decreases [21]. Also in this analysis, it was found that the conversion rate was higher in the group of patients operated on by residents compared to the group operated on by specialists, but this difference was not statistically significant (p>0.05).

The occurrence of conversion negatively affects the results of treatment [1,4]. Patients who underwent conversion have a longer duration of surgery, longer hospitalization, a higher incidence of surgical site infection, and a higher rate of clinically significant complications (Garde III and higher on the Clavien-Dindo scale) compared to patients whose procedure was performed laparoscopically [1,4,6,18,22]. These relationships were also confirmed in this analysis (p<0.0001). For this reason, it is important to determine the group of patients with increased risk of conversion, taking into account the deterioration of outcomes and the increase in costs associated with the treatment of symptomatic cholelithiasis if conversion occurs.

The conversion rate among patients undergoing LC is similar regardless of whether the ERAS protocol was used [23]. Our analysis also showed no such relationship. Additionally, the use of the ERAS protocol does not affect the duration of the surgical procedure [24]. It should be remembered that the extension of the surgical procedure time is an independent factor in the occurrence of clinically significant postoperative complications in gallbladder surgery [25].

The use of the ERAS protocol shortens the hospitalization time in the group of patients undergoing LC [24,26,27], which is also confirmed in our analysis. Shortening the hospitalization time significantly reduces the costs associated with the treatment of this group of patients [26]. The shortened length of stay in this group of patients is due, among other things, to faster mobilization after surgery.

Our analysis showed that in the group of patients in whom the ERAS protocol was used, the percentage of surgical site infections was lower, but the difference was not statistically significant. The lack of statistical significance may be due to the low percentage of surgical site infections in the studied groups. According to Akhtar et al., the use of the ERAS protocol does not increase the risk of surgical site infection after LC [26]. Importantly, it also does not increase the risk of readmission to the hospital after surgery due to possible postoperative complications [26].

In this analysis, no statistically significant difference was found in the occurrence of clinically significant postoperative complications depending on the use of the ERAS protocol. The use of the ERAS protocol does not increase the risk of clinically significant postoperative complications [28]. According to Zhang et al., the use of the ERAS protocol in gallbladder and biliary tract surgery does not affect the occurrence of clinically significant postoperative complications, such as intraoperative bleeding or bile leakage [29]. However, the use of this protocol reduces the frequency of complications such as postoperative nausea and vomiting [27,28]. The use of the ERAS protocol affects the level of postoperative pain experienced by the patient and reduces the use of opioid analgesics, thus also accelerating rehabilitation in the postoperative period [27,30]. Moreover, patients who have undergone ERAS have less anxiety related to surgery, which also improves treatment outcomes [30]. In our center, core ERAS elements include shortened preoperative fasting, early mobilization, standardized analgesia, and avoidance of drains. While ERAS is unlikely to directly influence the anatomical or inflammatory factors leading to conversion, optimized perioperative physiology may reduce the surgeon's threshold for persevering with laparoscopy.

These findings may guide surgeons in identifying patients at increased risk of conversion, enabling better intraoperative preparedness and setting realistic expectations for patients. Further studies are warranted to assess whether preoperative stratification based on these variables can optimize surgical outcomes.

An undoubted limitation of this study is its retrospective nature and the limited size of the analyzed population, taking into account the incidence of cholelithiasis. However, despite these limitations, it was possible to identify preoperative risk factors for conversion from laparoscopic to open surgery in the case of cholecystectomy performed due to symptomatic cholelithiasis.

## Conclusions

The most important preoperative risk factors for conversion in the case of cholecystectomy performed due to symptomatic cholelithiasis include male gender, older age, type 2 diabetes, and a history of an episode of AC. The occurrence of conversion is associated with the increased duration of surgery and hospitalization, a higher rate of surgical site infections, and a higher rate of clinically significant complications according to the Clavien-Dindo scale.

The use of the ERAS protocol in a group of patients undergoing LC due to symptomatic cholelithiasis improves short-term treatment outcomes. It is also safe, as it does not increase the conversion rate or clinically significant postoperative complications.
